# Factors Forming the Consumers’ Willingness to Pay a Price Premium for Ecological Goods in Ukraine

**DOI:** 10.3390/ijerph16050859

**Published:** 2019-03-08

**Authors:** Anatolii Kucher, Maria Hełdak, Lesia Kucher, Beata Raszka

**Affiliations:** 1 NSC Institute for Soil Science and Agrochemistry Research named after O.N. Sokolovsky 4, Chaikovska Street, 61024 Kharkiv, Ukraine; anatoliy_kucher@ukr.net; 2 V.N. Karazin Kharkiv National University, 4 Svobody Sq., 61022 Kharkiv, Ukraine; 3 Wroclaw University of Environmental and Life Sciences, Norwida 25, 50-375 Wrocław, Poland; beata.raszka@upwr.edu.pl; 4 Kharkiv National Agrarian University named after V.V. Dokuchaev, Education Campus KNAU, 62483 Kharkiv, Ukraine; kucher@knau.kharkov.ua

**Keywords:** ecological goods, consumers’ willingness, price premium, ecological economics, Ukraine

## Abstract

This study discusses the identification of factors affecting consumers’ willingness to pay a price premium for ecological goods. The study was carried out in selected regions of Ukraine, in the cities of Kharkiv and Kyiv. The study applied various research methods, in particular public opinion analysis based on conducted surveys and the statistical inference method. The conducted research may constitute the scientific basis for the assessment of this market segment development. The comparison of consumer attitudes, presented by the residents of major Ukrainian cities regarding environmental goods, revealed their willingness to pay a price premium depending primarily on the purchasing power of the population, but also on gender, age, and social status. The analysis of differences in the cross-tabulation of quality characteristics was performed using Pearson’s chi-square test, which showed that, for example, men were more willing than women to choose environmental products due to their environmental safety and their selection was more often than in case of women based on environmental goods’ price. The collected research results can be used to assess the development prospects of environmental goods’ market, to construct the set of measures increasing the willingness level of domestic consumers to pay a price premium for ecological products, and to take up decisions about the production of ecological goods.

## 1. Introduction

Along with the economic growth of countries and the increase of ecological awareness among populations, the situation of the market for environmental goods has been changing. The ongoing socio-economic transformations and the changes experienced in the natural environment affect the demand for environmentally friendly products.

Recent surveys [1] indicate using various terms in a synonymous way, in particular, the discussed products are referred to as: “ecological”, “green”, “sustainable”, “friendly to the environment”, “organic”, and “environmentally sound”. In our research the term “ecological goods” was used, which stands for economically efficient and environmentally friendly products, and allowed us to resolve the contradiction between economic growth and the guarantee of ecological safety [1,2].

In the current era, the concern for economic downturn, the reduction of environmental impacts, and sustainable development have become the major research subject taken up by numerous scientists, practitioners, and even industrial entities [3,4,5,6,7,8,9]. Urban sprawl impact on farmland conversion in suburban areas, the costs of urbanization, and foodsheds are the next examples of research topics for conducting environmental studies [10,11,12,13,14].

The willingness of households to pay a price premium for products in various parts of the world has been analysed by many scientists [15,16,17,18,19,20]. However, already in 2010, Aichlmayr [21] observed that: “In recent years, green consumption and marketing have triggered close attention and investments from producers”.

The willingness of US consumers to pay a price premium for ecological (“green”) products has been growing, even though relatively slowly in both the United States and in China. According to the recent research carried out by GfK (the company processing and analysing data of the world’s largest firms and leading brands), it was established that in 2017, a willingness to pay more for ecological products was declared by 56% of surveyed Americans, which shows an increase of 3% compared to 2010. In China, the annual growth rate goods and services of environmental reached 30%; in western countries, on average, goods and services of environmental purpose spending went up to 10% of the family budget, the demand for them is increasing; and the growth rate of the environmental market went up to 8% per year in the Baltic States, and to 10% in Canada [22].

Further research revealed that many consumers are prepared to pay a small price premium for ecological goods; however, the willingness to pay declines along with the premium increase [3]. A survey covering 1000 consumers in Europe and the United States showed that over 70% of them while purchasing such goods as cars, construction materials, electronics or furniture would pay an additional 5% for a green product if it met the same standards as an alternative one. However, less than 10% of consumers said they would choose “green” products if the price premium increased up to 25% [23,24]. Willingness to pay, which denotes the maximum price that a consumer is willing to pay for a particular or a bundle of products, plays a decisive leverage on their choice behaviour [25].

In Ukraine, the study of consumers’ willingness to pay a price premium for ecological goods of various types was initiated and conducted by scientists from the Sumy Scientific School, in particular: Prokopenko [26,27], Iliashenko [1,28,29,30], Kuchmiov [31], Kucher [32], and others. The study confirmed that a significant proportion of respondents remain highly willing to purchase ecological goods, even at a higher price, provided the price rise does not exceed the limits allowed by consumers, taking into account their income level.

The production costs of eco-labelled products are higher than those of conventional ones because eco-labelled products require careful management from the raw materials and subsidiary materials to the packaging (the product is manufactured using an eco-friendly process and production method) [33,34].

It was also found that consumers are willing to pay an average additional price premium for improving the environmental properties of goods in the amount of 9.9% [31]. The discussed research was carried out in the Ukrainian city of Sumy, populated by approximately 269,000 residents. Therefore, the authors conducted the research in more locations to extend and explore the situation characterising consumers’ willingness to pay ecological goods on the Ukrainian scale.

The purpose of the study is to identify factors affecting consumers’ willingness to pay a price premium for ecological goods. The public opinion survey was carried out in Ukraine, in the cities of Kharkiv and Kyiv.

## 2. Materials and Methods

The presented study aims at identifying the factors affecting consumers’ willingness to pay a price premium for ecological goods depending on their age, gender or social status. A general understanding of “premium” is that it commands a higher than normal price relative to normal goods.

The following hypotheses were put forward in the article:

**Hypothesis** **N_1_:** *Ecological awareness manifested through the willingness to buy a product and the indication of current problems on the market of environmental goods increases along with the age of the analysed group of Ukrainian citizens.*

**Hypothesis** **N_2_:** *The respondents are characterised by diverse preferences when purchasing ecological goods depending on their gender and age.*

The study also takes into account the respondents’ opinions regarding problems on the market of ecological environmental goods depending on social status.

The research stages:

- The subject literature review and the research objective formulation,
- Developing the questionnaire (survey),
- Conducting surveys in the selected cities,
- The analysis of collected results using the descriptive method and the statistical inference method,
- The identification of factors affecting consumers’ willingness to pay a price premium and the verification of the research theses.

Questionnaire: Research on “The different groups of local consumers’ willingness to pay price premiums for ecological goods” added below (Table 1).

The research covered the cities of Kharkiv and Kiev representing, at the same time, one of the largest cities in Ukraine. Kharkiv is located in the eastern part of Ukraine and is populated by approximately 1.4 million inhabitants, whereas Kiev is placed in the north-central part of the country with a population of about 2.9 million residents.

The survey covered 200 people, all residents of Ukraine. Half of the respondents (50%) originated from Kiev, and the other half (50%) from Kharkiv. Women constituted the vast majority of the respondents—71% (almost three-quarters of the surveyed population). Men, in turn, accounted for over a quarter of the respondents (29%). The majority of the surveyed population was under the age of 30—69% (over two-thirds of the respondents). A similar percentage of the respondents was represented by the population aged between 31 and 40 and between 41 and 50—14.5% and 11%, respectively. The least numerous group covered the people aged over 50, who accounted for 5.5% of the respondents. The largest group among those surveyed was made up of manual labourers—49.5% (almost half of the respondents). The learning population (pupil/student) constituted nearly one-third of the respondents (32%), whereas a similar percentage of the surveyed population did not work professionally (unemployed) or had their own business (entrepreneurs)—8.5% and 7%, respectively. In addition, the survey also covered pensioners (2.5%) and office workers (0.5%)—Table 2, Table 3 and Table 4.

The authors adopted the following explanatory variables: (1) gender (female vs. male), (2) age (aged less than 30 vs. 31–40 vs. 41–50 vs. over 50 years of age), (3) social status (manual labourer vs. entrepreneur vs. unemployed vs. pupil/student vs. pensioner); and explained variables: (1) the indication of reasons for ecological goods’ preferences (4 variables); (2) the specification of factors most important in the selection of ecological goods (6 variables); (3) using specific ecological goods (6 variables); (4) assessing the need for further development of ecological production in Ukraine (4 variables); and (5) the identification of current problems on the market of ecological goods (4 variables). The analysis of differences in the cross-tabulation of quality characteristics was performed using Pearson’s chi-square test (*χ*^2^ independence test). In all conducted analyses, the maximum permissible type I error *α* = 0.05 was adopted, whereas *p* ≤ 0.05 was considered statistically significant.

## 3. Results and Discussion

For the purposes of checking whether women differed from men in terms of indicating the reasons underlying their preferences of ecological goods, the chi-square independence test was carried out. The table below shows the obtained research results (Table 5).

**Figure 1:**
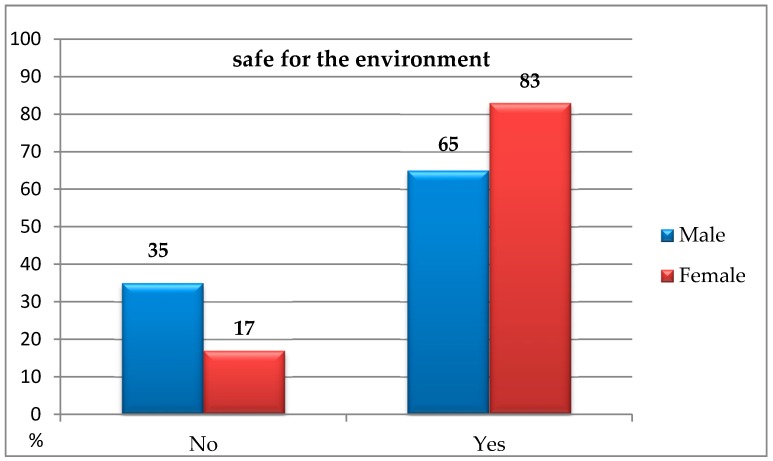
Preference of ecological goods due to their environmentally friendly aspect among people of different gender.

The analyses carried out using the chi-square independence test indicated the statistically significant differences between the studied groups in terms of the environmentally friendly goods: *χ*^2^(1, *N* = 200) = 5.90; *p* < 0.050; *ϕ* = 0.17—it means that men, more often than women, choose ecological goods due to their environmentally friendly aspect (Figure 1).

In terms of the other variables, no statistically significant differences were observed between the analysed groups (*p* > 0.050). It means that women did not differ from men regarding the identification of other preferences for choosing ecological products (i.e., high quality, positive impact on health, and popularity/current trends).

In order to check whether women differed from men in terms of specifying the most important factors while selecting ecological goods, the chi-square independence test was applied again in the course of conducted analyses. According to Kumakawa [35], an important topic for research on environmental evaluation is gender differences in willingness to pay for environmental goods. The table below shows the obtained research results (Table 6).

**Figure 2:**
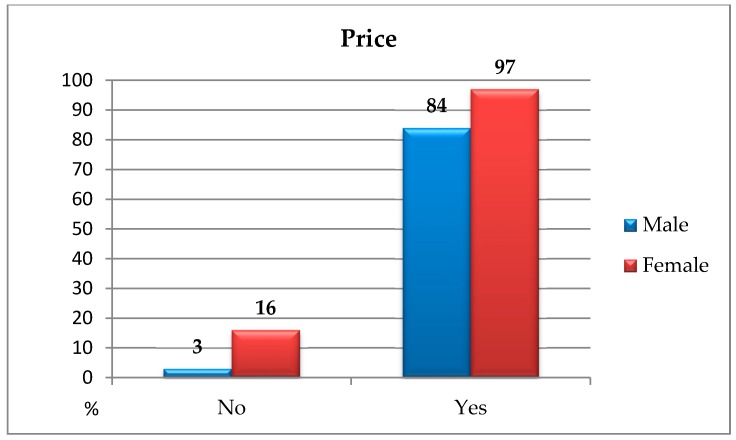
Price as the important criterion when choosing environmental goods among people of a different gender.

The analyses carried out using the chi-square independence test indicated the statistically significant differences between the studied groups in terms of price: *χ^2^*(1, *N* = 200) = 6.12; *p* < 0.050; *ϕ* = 0.17. It means that men more often than women were guided by the price factor when choosing ecological goods (Figure 2).

While Dupont [36] suggested that women are willing to pay more to avoid environmental health risks for their children, Dietz et al. [37] report that their results show that women have stronger environmental beliefs but are less willing to sacrifice for environmental protection. The results of the conducted tests are reversed. Carlsson and al. [38] suggest that male respondents have a higher willingness to pay than female respondents for ecological and fair-trade coffee. Maybe it depends on ecological goods, which should be investigated.

In terms of the other variables, no statistically significant differences were observed between the analysed groups (*p* > 0.050). This means that women did not differ from men regarding the identification of other factors influencing their choice of environmental products (i.e., manufacturer/brand, the presence of certification marks, packaging design, taste, and storage method).

Consumers have higher percentages of willingness to pay for environmentally friendly, low-priced goods, compared to environmentally friendly, high-priced goods [35,39].

The research also covered respondents’ age and its impact on using ecological products. The table below shows the obtained research results related to checking whether the individuals representing various age groups differed in terms of using specific environmental goods (Table 7).

The analyses carried out using the chi-square independence test indicated the statistically significant differences between the studied groups in terms of:

- cosmetics: *χ*^2^(3, *N* = 200) = 13.87; *p* < 0.010; *V* = 0.27—it means that the respondents aged 31–40 were more likely to use ecological cosmetics than the ones representing other age groups, whereas people aged over 50 were less likely to use such products compared to younger age groups;
- packaging and bags: *χ*^2^(3, *N* = 200) = 14.36; *p* < 0.010; *V* = 0.28—it means that the respondents aged 31–40 used ecological packaging and bags more often than the ones in other age groups, whereas people aged over 50 used such products less often compared to younger age groups.

In terms of the other variables, no statistically significant differences were identified between the analysed groups (*p* > 0.050). It means that the respondents from different age groups did not differ regarding the usage of other environmental products (i.e., food, clothes, furniture, and ecological supplements).

The survey also referred to the need for further development of ecological production in Ukraine according to the assessment of people representing different age groups. The conducted analyses showed the statistically significant differences between the groups in terms of the current unsatisfactory food quality: *χ*^2^(3, *N* = 200) = 8.35; *p* < 0.050; *V* = 0.22. This means that the respondents aged 31–40 confirmed the need for further development of ecological production in Ukraine less often than people in other age groups due to the insufficient quality of modern food products.

In order to check whether people presenting various social statuses differed from each other in terms of indicating current problems on the market of environmental goods, the chi-square independence test was carried out. Due to the small size of the “office worker” group (1 respondent), it was excluded from further analysis. The analyses showed statistically significant differences between the studied groups regarding all analysed variables.

**Figure 3:**
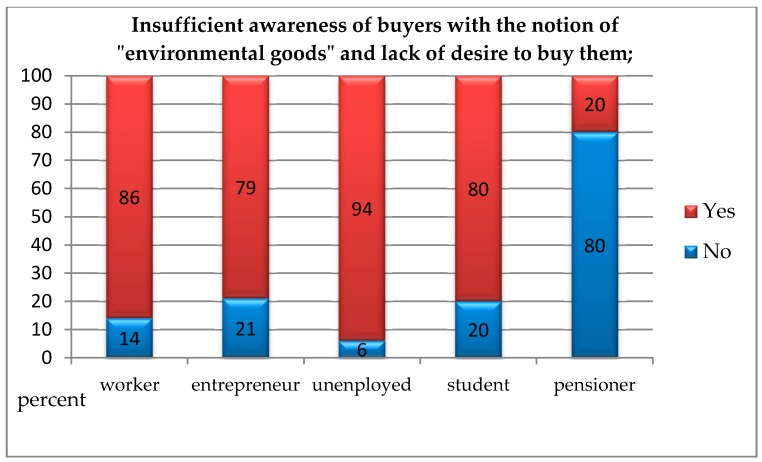
Insufficient awareness of consumers regarding the concept of “ecological goods” and the lack of willingness to purchase them as the current problem with the market for environmental goods according to people with a different social status.

**Figure 4:**
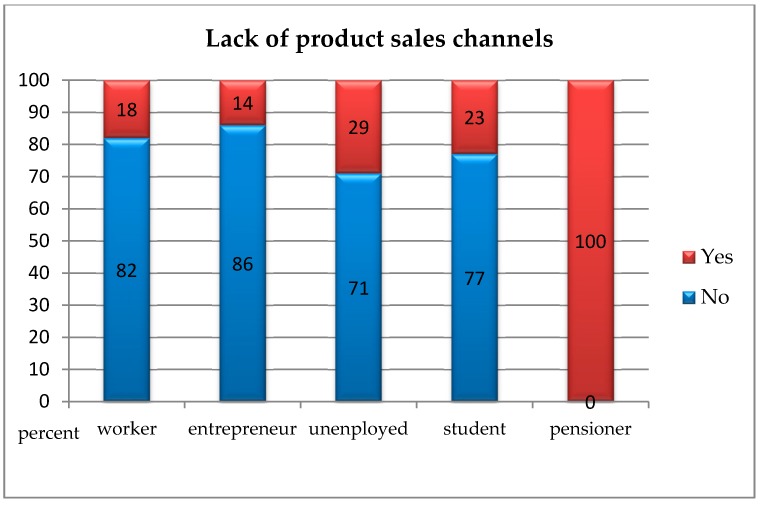
The absence of sales channels as a current problem with the market for environmental goods according to people with a different social status.

**Figure 5:**
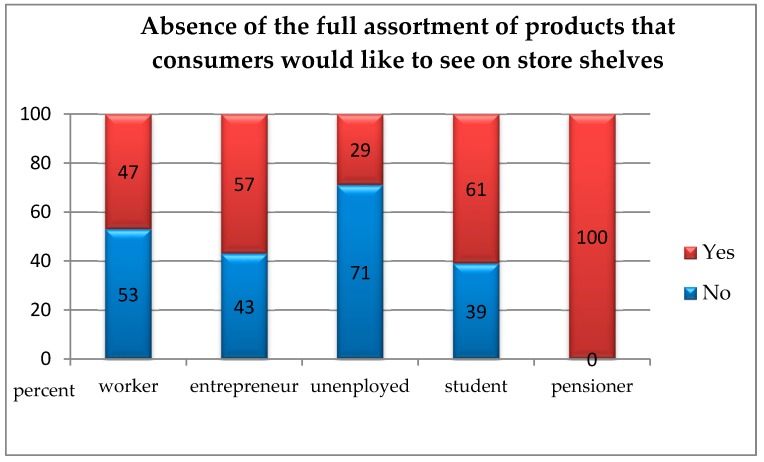
The absence of the full range of products which consumers would like to see on store shelves as a current problem with the market for environmental goods according to people with a different social status.

**Figure 6:**
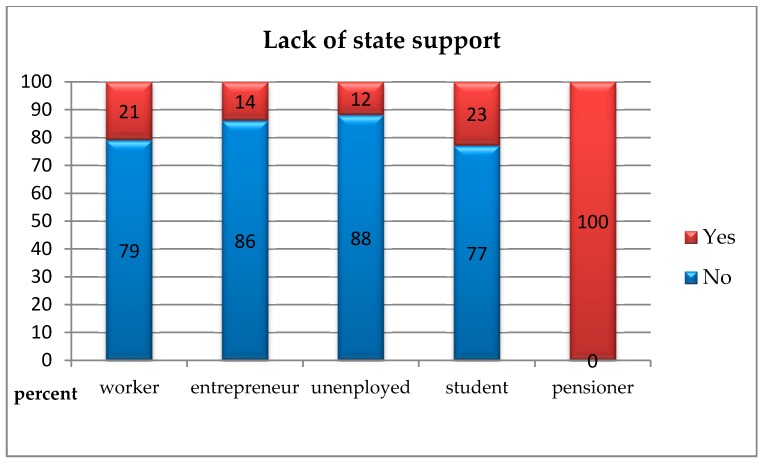
The absence of state support as a current problem with the market for environmental goods according to people with a different social status.

- Insufficient awareness of consumers regarding the concept of “ecological goods” and the lack of willingness to purchase them: *χ*^2^(4, *N* = 199) = 12.29; *p* < 0.050; *V* = 0.29. This means that pensioners less often than manual labourers, entrepreneurs, unemployed, and pupils/students pointed to the insufficient awareness of customers regarding “ecological goods” and the lack of willingness to purchase them as a current problem with the market for environmental goods (Figure 3);
- The absence of sales channels for products: *χ*^2^(4, *N* = 199) = 15.21; *p* < 0.010; *V* = 0.31. This means that pensioners more often than manual labourers, entrepreneurs, unemployed, and pupils/students pointed to the absence of sales channels for products as a current problem with the market for environmental goods (Figure 4);
- The absence of the full range of products which consumers would like to see on store shelves: *χ^2^*(4, *N* = 199) = 10.90; *p* < 0.050; *V* = 0.24. This means that pensioners more often than manual labourers, entrepreneurs, unemployed, and pupils/students pointed to the absence of sales channels for products as a current problem with the market for environmental goods, whereas the unemployed identified this problem less often than others (Figure 5);
- The absence of state support: *χ*^2^(4, *N* = 199) = 14.52; *p* < 0.010; *V* = 0.31. This means that pensioners more often than manual labourers, entrepreneurs, unemployed, and pupils/students pointed to the absence of state support products as a current problem with the market for environmental goods (Figure 6).

According to Wang et al. [40,41,42], studies have shown that age, income, education, regional difference, and attitude towards certified traceability systems were significant factors affecting consumers’ attitudes towards a price premium for certified food.

## 4. Conclusions

The conducted research allowed for partial verification of the presented thesis that: ecological awareness expressed through the willingness to purchase a product and the indication of current problems with the market for environmental goods increases along with the age of the analysed group of Ukrainian citizens.

The willingness to purchase ecological products (cosmetics, packaging, and bags) was reported more frequently by people aged between 31 and 40, whereas those over 50 years of age were less likely to use such products than people in younger age groups. In turn, pensioners less frequently than manual labourers, entrepreneurs, unemployed, and students indicated insufficient awareness of customers regarding “ecological goods” and the lack of willingness to buy them as the current problem with the market for environmental goods. Also, older people (pensioners) more often than other professional groups identified the absence of product sales channels and the absence of state support as a current problem with the market for economics goods. Post-working age individuals do notice the problem of providing sufficient information and product promotion as well as state involvement.

Zhou [43] conducted research on the safety cognition and purchasing behaviour of urban residents in Zhejiang Province (China), and the results showed that the educational background, family structure, and consumers’ concern about vegetable safety were significantly correlated with consumers’ cognition of safe vegetable consumption [44].

The second Hypothesis (N_2_) was proven in this article: the respondents were characterized by different preferences when purchasing ecological products depending on their gender and age. The conducted analyses showed that men were more likely than women to choose ecological goods because of their environmental safety and were more often than women guided by the purchase price when choosing ecological goods.

The conducted research can be used to improve state policy in the promotion of ecological goods and the policy of companies manufacturing products using environmentally friendly technologies. A statement can be risked that “being green” needs time and space in peoples’ lives that is not available in increasingly busy lifestyles [45].

## Figures and Tables

**Table 1:** Questionnaire: The different groups of local consumers’ willingness to pay price premiums for ecological goods.

**QUESTIONNAIRE** Research on: **“The different groups of local consumers’ willingness to pay price premiums for ecological goods”**
Welcome to you! We invite you to take place in our survey, that we are conducting for revealing local consumers’ willingness to pay price premiums for ecological goods. You will be asked a few questions. Please select an answer that corresponds to your position. This questionnaire is anonymous, the data obtained in a generalized form will be used for scientific and practical purposes
**1.** **Gender:** ○ male; ○ female. **2.** **Age:** ○ under 30; ○ 31–40; ○ 41–50; ○ over 50. **3.** **Education:** ○ general secondary; ○ specialized secondary; ○ uncompleted higher; ○ higher; ○ Doctor of Philosophy/Doctor of Science. **4.** **Social status:** ○ worker; ○ official; ○ entrepreneur; ○ unemployed; ○ student; ○ pensioner. **5.** **Are you satisfied with the quality of the products you buy?** ○ yes; ○ no. **6.** **When you make a purchase, do you choose environmentally friendly products?** ○ yes; ○ no. **7.** **What product do you consider environmental?** ○ the one that I bought in the village; ○ the one that I cultivate myself; ○ with special markings on the package and the certificate number; ○ with green leaves, inscriptions BIO, ORGANIC, ECO; ○ any good product (without preservatives, GMO, etc.). **8.** **Indicate the reasons why you prefer ecological products?** ○ high-quality goods; ○ good for health; ○ safe for the environment; ○ it’s fashionable. **9.** **What do you pay attention to when choosing environmental products?** ○ producer; ○ price; ○ the presence of certification marks; ○ appearance of packaging; ○ taste; ○ storage. **10.** **Where do you usually buy environmental products?** ○ in hypermarkets/supermarkets; ○ in specialized stores; ○ the Internet; ○ I order from the catalogue, from the consultants; ○ in the market/from hands; ○ in drugstores. **11.** **What kind of environmental products do you use?** ○ cosmetics; ○ food; ○ clothes; ○ furniture; ○ packets and bags; ○ supplements. **12.** **How many percent are you willing to pay for environmental goods in comparison with ordinary products?** ○ less than 10%; ○ 10–25%; ○ 25–50%; ○ 50–100%; ○ larger than 100%. **13.** **How much money you are willing to spend on environmental goods in a month?** ○ up to 200 UAH currency of Ukraine; ○ 200–500 UAH; ○ 500–1000 UAH; ○ as much as it will take for a proper nutrition; ○ not ready to spend extra money on environmental products. **14.** **How, in your opinion, it is necessary to stimulate the demand for environmental goods in Ukraine?** ○ affordable prices; ○ advertising in the media; ○ availability in the trading network; ○ the development and approval of the legislative and regulatory framework. **15.** **Is there a need for further development of ecological production in Ukraine?** ○ so, the consumption of environmentally friendly products will ensure a high quality of life; ○ yes, because nowadays the quality of food is unsatisfactory; ○ so, for the sake of future generations; ○ no, the consumer is now sufficiently supplied with food. **16.** **What issues, in your opinion, are currently in the market for environmental goods?** ○ insufficient awareness of buyers with the notion of “environmental goods” and lack of desire to buy them; ○ lack of product sales channels; ○ absence of the full assortment of products that consumers would like to see on store shelves; ○ lack of state support.
**Thank you for participating in the study!**

**Table 2:** Gender structure in the sample (*N* = 200).

Gender	Number	% of Total
Male	58	29.00
Female	142	71.00

**Table 3:** Age structure of the sample (*N* = 200).

Age	Number	% of Total
under 30	138	69.00
31–40	29	14.50
41–50	22	11.00
over 50	11	5.50

**Table 4:** Social status structure in the sample (*N* = 200).

Social Status:	Number	% of Total
Worker	99	49.50
Official	1	0.50
Entrepreneur	14	7.00
Unemployed	17	8.50
Student	64	32.00
Pensioner	5	2.50

**Table 5:** Gender vs. the indication of preferences for ecological goods—*χ*^2^ independence test.

Indicate the Reasons Why You Prefer Ecological Products?		Gender				Together		*χ* ^2^	*p*
		Male		Female					
		*n*	% From Group	*n*	% From Group	*n*	% of Together		
high-quality goods	No	11	18.97	19	13.38	30	15.00	1.01	0.315
	Yes	47	81.03	123	86.62	170	85.00		
	Total:	58	100.00	142	100.00	200	100.00		
good for health	No	0	0.00	0	0.00	0	0.00	no variability	
	Yes	58	100.00	142	10.00	200	100.00		
	Total:	58	100.00	142	100.00	200	100.00		
safe for the environment	No	10	17.24	49	34.51	59	29.50	5.90	0.015
	Yes	48	82.76	93	65.49	141	70.50		
	Total:	58	100.00	142	100.00	200	100.00		
it’s fashionable	No	32	55.17	97	68.31	129	64.50	3.10	0.078
	Yes	26	44.83	45	31.69	71	35.50		
	Total:	58	100.00	142	100.00	200	100.00		

Note: *n*—size; *χ*^2^—chi-square independence test result; *p*—significance level.

**Table 6:** Gender vs. the specification of factors most important in the selection of ecological goods of factors—*χ*^2^ independence test.

What Do You Pay Attention to When Choosing Environmental Products?		Gender				Together		*χ^2^*	*p*
		Male		Female					
		*n*	% From Group	*n*	% From Group	*n*	% of Together		
Producer	No	49	84.48	124	87.32	173	86.50	0.28	0.594
	Yes	9	15.52	18	12.68	27	13.50		
	Total:	58	100.00	142	100.00	200	100.00		
Price	No	2	3.45	23	16.20	25	12.50	6.12	0.013
	Yes	56	96.55	119	83.80	175	87.50		
	Total:	58	100.00	142	100.00	200	100.00		
The presence of certification marks	No	7	12.07	28	19.72	35	17.50	1.67	0.196
	Yes	51	87.93	114	80.28	165	82.50		
	Total:	58	100.00	142	100.00	200	100.00		
Appearance of packaging	No	36	62.07	99	69.72	135	67.50	1.10	0.295
	Yes	22	37.93	43	30.28	65	32.50		
	Total:	58	100.00	142	100.00	200	100.00		
Taste	No	14	24.14	41	28.87	55	27.50	0.46	0.496
	Yes	44	75.86	101	71.13	145	72.50		
	Total:	58	100.00	142	100.00	200	100.00		
Storage	No	13	22.41	22	15.49	35	17.50	1.37	0.242
	Yes	45	77.59	120	84.51	165	82.50		
	Total:	58	100.00	142	100.00	200	100.00		

Note: *n*—size; *χ*^2^—chi-square independence test result; *p*—significance level.

**Table 7:** Age vs. using specific environmental goods—*χ*^2^ independence test.

What Kind of Environmental Products Do You Use?		Age								Total		χ^2^	*p*
		Under 30		31–40		41–50		Over 50					
		*n*	% From Group	*n*	% From Group	*n*	% From Group	*n*	% From Group	*n*	% of Total		
Cosmetics	No	99	71.74	13	44.83	17	77.7	11	100.00	140	70.00	13.87	0.002
	Yes	39	28.26	16	55.17	5	22.73	0	0.00	60	30.00		
	Total:	138	100.00	29	100.00	22	100.00	11	100.0	200	100.00		
Food	No	15	10.87	5	17.24	5	22.73	0	0.00	25	12.50	4.25	0.219
	Yes	123	89.13	24	82.76	17	77.27	11	100.00	175	87.50		
	Total:	138	100.00	29	100.00	22	100.00	11	100.00	200	100.00		
Clothes	No	138	100.00	29	100.00	22	100.00	11	100.00	200	100.00	no variability	
	Yes	0	0.00	0	0.00	0	0.00	0	0.00	0	0.00		
	Total:	138	100.00	29	100.00	22	100.00	11	100.00	200	100.00		
Furniture	No	138	100.00	29	100.00	22	100.00	11	100.00	200	100.00	no variability	
	Yes	0	0.00	0	0.00	0	0.00	0	0.00	0	0.00		
	Total:	138	100.00	29	100.00	22	100.00	11	100.00	200	100.00		
Packets and bags	No	107	77.54	14	48.28	18	81.82	11	100.00	150	75.00	14.36	0.002
	Yes	31	22.46	15	51.72	4	18.18	0	0.00	50	25.00		
	Total:	138	100.00	29	100.00	22	100.00	11	100.00	200	100.00		
Supplements	No	127	92.03	25	86.21	22	100.00	11	100.00	185	92.50	3.35	0.292
	Yes	11	7.97	4	13.79	0	0.00	0	0.00	15	7.50		
	Total:	138	100.00	29	100.00	22	100.00	11	100.00	200	100.00		

Note: *n*—size; *χ*^2^—chi-square independence test result; *p*—significance level.
